# Precise insertion and guided editing of higher plant genomes using Cpf1 CRISPR nucleases

**DOI:** 10.1038/s41598-017-11760-6

**Published:** 2017-09-14

**Authors:** Matthew B. Begemann, Benjamin N. Gray, Emma January, Gina C. Gordon, Yonghua He, Haijun Liu, Xingrong Wu, Thomas P. Brutnell, Todd C. Mockler, Mohammed Oufattole

**Affiliations:** 1 Benson Hill Biosystems, 1100 Corporate Square Dr, St. Louis, MO 63132 USA; 2 Donald Danforth Plant Science Center, 975N, Warson Road, St. Louis, MO 63132 USA

## Abstract

Precise genome editing of plants has the potential to reshape global agriculture through the targeted engineering of endogenous pathways or the introduction of new traits. To develop a CRISPR nuclease-based platform that would enable higher efficiencies of precise gene insertion or replacement, we screened the Cpf1 nucleases from *Francisella novicida* and *Lachnospiraceae bacterium ND2006* for their capability to induce targeted gene insertion via homology directed repair. Both nucleases, in the presence of a guide RNA and repairing DNA template flanked by homology DNA fragments to the target site, were demonstrated to generate precise gene insertions as well as indel mutations at the target site in the rice genome. The frequency of targeted insertion for these Cpf1 nucleases, up to 8%, is higher than most other genome editing nucleases, indicative of its effective enzymatic chemistry. Further refinements and broad adoption of the Cpf1 genome editing technology have the potential to make a dramatic impact on plant biotechnology.

## Introduction

Plant biotechnology using traditional molecular biology and transformation technologies has resulted in crops that have reduced energy intensive inputs, improved yields, and increased food, fuel, and fiber security across the globe. New advances in biotechnology, namely genome editing, have the potential to dramatically increase plant yields and bring product concepts to market that previously had large technical and/or regulatory barriers to market entry^1^. The adoption of genome editing in industrial plant biotechnology has resulted in new products with improved disease tolerance, yield, and food quality^2–4^. These advanced products were developed at a fraction of the time and cost of traditional ag-biotech traits. Broad adoption of this technology along with advances in genomics and breeding will result in innovation across the spectrum of native and novel traits including targeted allele swapping, trait stacking, and modification of expression elements of native genes^5^. The development of an easily accessible and functionally efficient genome editing platform will result in a new age of plant biotechnology.

Plant genomes can be edited using a variety of technology platforms including meganucleases, TALENs, CRISPR-Cas9, and zinc finger nucleases^6, 7^. Development of the CRISPR-Cas9 platform dramatically decreased the cost of performing genome editing experiments and allowed for expanded use of the technology in the plant sciences^8^. While easier to implement than TALENs, zinc fingers, and meganucleases, CRISPR-Cas9 has raised some concerns with off-target effects, and its uncertain intellectual property landscape also has limited the broad adoption of CRISPR-Cas9 technology^9, 10^. Recently, an alternative family of CRISPR nucleases, Cpf1, has been identified and shown to function in editing the genome of human cells^11^. Cpf1 enzymes are a family of type V CRISPR nucleases that includes both endoribonuclease and endodeoxyribonuclease activities, allowing these nucleases to both process CRISPR-RNAs (crRNAs) and generate double strand breaks of DNA respectively^12^. The dual enzymatic activities allow for multiplex targeting from a single crRNA transcript^13^. In addition, Cpf1 nucleases have also been shown to have lower rates of off-target edits relative to Cas9 nucleases^14, 15^.

Cpf1 nucleases in combination with crRNAs have been shown to generate indel mutations via non-homologous end joining repair (NHEJ) in both prokaryotic and eukaryotic systems^11, 16^. Recently, several of these Cpf1 nucleases were shown to generate indel mutations in both monocot and dicot plant species^17, 18^. While NHEJ mediated modifications have significant value for plant genome editing, enabling homology directed repair (HDR) modifications in either plant or animal genomes would better expand the utility of this technology to even far more impactful applications, such as the creation of superior alleles through site specific mutations and targeted insertion of genes and/or regulatory elements.

The Cpf1 nucleases from *Fransicella novicida* (FnCpf1) and *Lachnospiraceae bacterium ND2006* (LbCpf1) were selected for investigation in plants with the goal of identifying CRISPR nuclease activities, when combined with the plant endogenous repairing events, that lead to gene knockouts and targeted insertion, both of which are inherently important to a robust plant genome editing platform. As previously described, LbCpf1 was able to generate indels in both *Escherichia coli* (*E. coli*) and human HEK293 cells, while the success of FnCpf1 was limited to *E. coli* ^11^. Due to the inconsistencies of host specific performance of previously described genome editing nucleases^19^, it was not clear either that negative or positive results in one system, whether *in vitro*, eukaryotic, or prokaryotic, would reliably predict performance in another system. These inconsistencies are the results of the complex nature of heterologous expression of a genome editing system, which may result from suboptimal codon usage, the efficacy of nuclear localization tags, variable expression levels of each editing component, crRNA stability and other variables^20^. In our experiments, multiple Cpf1 nucleases were tested in our plant transformation system and both HDR and NHEJ repair genome edits were observed.

## Results and Discussion

### Cpf1 mediated targeted insertion vector design

**Figure 1:**
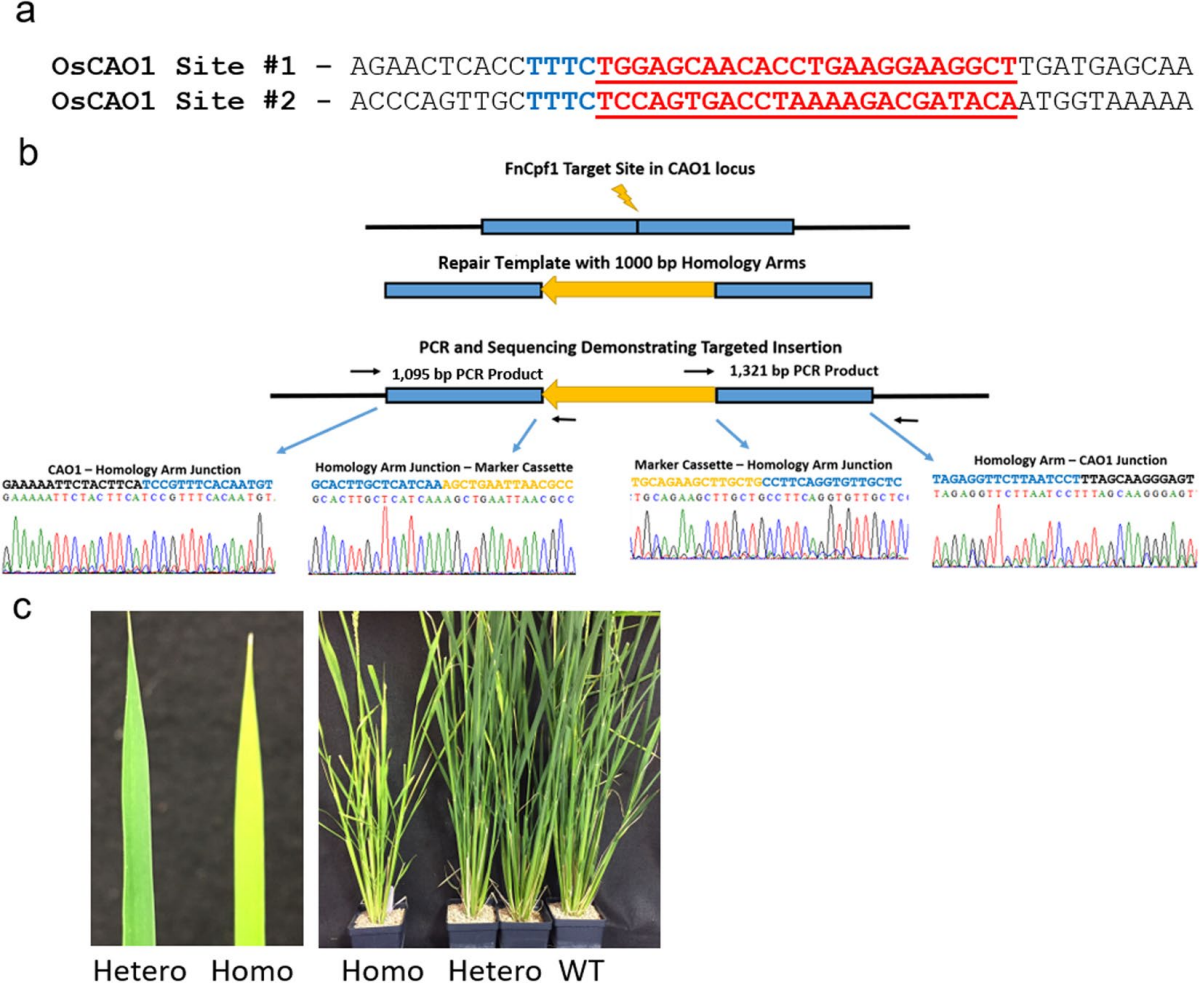
Targeted integration of hpt gene into the *CAO1* gene of rice. (**a**) Target sites for crRNA design. Site #1 is shown as the reverse complement for clarity. The PAM site is identified in blue and the target sequence is in red. (**b**) Schematic and sequence alignment of hpt insertion (yellow sequence) at *CAO1* site #1 using FnCpf1. Primers used for insertion screening are labeled P1 = #1Up Fwd (Table S1), P1 = 1Up Rev, P3 = Dwn Fwd, P4 = Dwn Rev. (**c**) Comparison of leaves and whole plants of homozygous *CAO1* (Homo) and heterozygous (Hetero) or wild type (WT) lines.

To test the capability of each Cpf1 enzyme to generate targeted gene insertion via HDR, a screen was developed that would result in a visual phenotype upon genome editing. The Chlorophyllide-*a* oxygenase gene of rice (*CAO1*) was selected as a target gene due to an easily visualized “yellow” phenotype in homozygous *CAO1* deletion plants, for loss-of-function of the *CAO1* gene results in the inability to convert chlorophyll *a* to chlorophyll *b* ^21^. In our experimental design, we included a hygromycin phosphotransferase gene (hpt), an antibiotic resistance marker, in the repair template (Fig. 1) that would both interrupt the rice *CAO1* coding sequence and expedite genetic screening (see details of primers used for screening (Table S1) and plasmid constructs used in this study (Table S2). In each transformation experiment, rice (*Oryza sativa* cv. Kitaake) calli were bombarded with a set of three plasmids (Table S3) containing a Cpf1 expression cassette, a crRNA expression cassette specific to each Cpf1 enzyme, and a repair template with an hpt gene under the control of the maize ubiquitin promoter flanked by 1 kb regions of rice genomic DNA known as homology arms (Figs 1b and S3). The Cpf1 genes, codon optimized for monocot plants, were driven by the 2 × 35 S CaMV promoter (Table S2). A nuclear localization signal tag (amino acid sequence: PKKKRKV, SV40 Large T-antigen) was included in the N-terminus of each Cpf1 protein. Target sequences within either exon 1 or exon 4 of the *CAO1* gene were tested indepenedently for target integration, both containing a TTTC PAM site, consistent with the PAM site requirements described previously for these enzymes^11^ (Figs 1a and S1, S4). These sites are unique within the rice genome and were designed to minimize off-target effects. For the complete design of biolistic transformation expression systems and repair templates (DNA sequences), please refer to our Supplementary Fig S1–S6.

### FnCpf1 mediated targeted insertions detected in rice callus

A set of three plasmids (131272, 131608, 131760, Tables S2 and S3) specific to FnCpf1 and *CAO1* site #1 (Experiment GE0001, Table S3) were bombarded into rice calli and hygromycin resistant calli were identified. Fragments of resistant calli were screened via polymerase chain reaction (PCR) for the presence of a targeted integration of the hpt resistance marker into the *CAO1* gene via HDR. Primer sequences used in these experiments can be found in Table S1. PCR assays were developed to amplify each junction fragment from the genomic region outside the homology arm into the resistance marker (Fig. 1b), In this case, amplification would only occur in the presence of an effective targeted integration event, since the primer sequences, e.g., those of P1 and P4, are only present in the genome but absent in our repairing piece (Fig. 1b). Out of 36 independent callus lines, 3 generated PCR products for both the upstream and downstream junctions, for a targeted integration frequency of 8% (Fig. S7 and Table 1). PCR fragments from callus line number 6 (Fig. S7, GE0001–6) were sequenced and aligned to the predicted insertion sequence (Fig. 1b). These alignments demonstrated a clean junction between the genomic DNA, homology arms, and both ends of the resistance marker. PCR analysis also demonstrated the presence of the hpt resistance marker and FnCpf1 cassette stably integrated in the genome of the transgenic lines (primers listed in Table S1). Sequencing of additional PCR fragments from independent callus lines GE0001-1 and GE0001-21 demonstrated identical results of precise targeted integration events (Fig. S8). In brief, all independently identified targeted insertions resulted in precise genomic site integration events, leaving intact junction regions between genomic DNA and upstream homology arm DNA, and downstream homology arm and corresponding genomic DNA.

**Table 1:** Results of Rice Biolistics Experiments.

Experiment	Target	Nuclease	Resistant Calli	Insertion Frequency	Indels Frequency
GE0001	OsCAO1 Site 1	FnCpf1	36	3 (8%)	1 (3%)
GE0001-R	OsCAO1 Site 1	FnCpf1	96	8 (8%)	31 (32%)
GE0031	OsCAO1 Site 1	LbCpf1	55	0 (0%)	1 (2%)
GE0046	OsCAO1 Site 2	LbCpf1	96	3 (3%)	10 (10%)

### Regenerated plants maintained the insertion and generated a phenotype in the T1 generation

Upon transfer to regeneration medium, callus line (GE0001-1) produced four sibling plantlets (T0), with each plantlet PCR positive for both the upstream and downstream junction elements (Fig. S9). The T1 seeds were collected individually from each putative transgenic T0 plant. The T1 seeds from each transgenic plant were germinated and screened for the presence of the hpt resistance marker using Quantitative PCR (qPCR). T1 seeds from GE0001-1.3 were planted and approximately 25% of seedlings were visually pale green, indicative of 3:1 Mendelian segregation. Upon growth in the greenhouse, T1 plant GE0001-1.3.1 was found to be homozygous for insertion via qPCR. As expected, this line produced a “yellow” leaf phenotype (Fig. 1c) and is absent of Chlorophyll *b* (Fig. S10) after pigments spectra analysis, consistent with a loss of function of the *CAO1* gene^21^.

To determine if FnCpf1 could introduce mutations outside of the target site, i.e., off-target effects, a preliminary analysis was performed to identify off-target cutting. Cas-OFFinder^22^ online software was used to identify the potential off-target sites within the rice genome. Two sites were identified with threshold setting of a 4 bp mismatch. Both genomic regions were amplified via PCR from multiple GE0001 events as well as from wild type rice genomic DNA. These fragments were subcloned, sequenced via Sanger sequencing and compared to the wild type sequences. No off-target mutations were detected at these genomic loci (data not shown).

### FnCpf1 mediated editing results in a high rate of HDR

The FnCpf1 targeted integration experiment into *CAO1* site #1 was replicated (GE0001-R, Table S3) on a larger scale of transformation screening to demonstrate the efficacy of the FnCpf1 nuclease to generate targeted gene insertion and to test the reproducibility of the system (Table 1). This experiment resulted in consistent frequency of targeted insertion (8%). This rate of targeted insertion is higher than other published experiments using the *S. pyogenes* Cas9 enzyme in plants^2, 23–25^. Under two reported experimental conditions in monocots, insertion rates of 2.5%-4.1% and 2.0%-2.2% were reported in maize and rice, respectively^23, 25^. Note that the maize experiments also include co-transformation of the ODP2 and WUS genes, which have been shown to dramatically increase the rate of transformation^23^. The higher rates of HDR demonstrated in our work may be attributed to the 5′ single stranded DNA ends that result from Cpf1 nuclease activity. Recently it was shown that the presence of 5′ single stranded DNA results in significantly increased rates of HDR relative to those achieved in the presence of blunt ends^26^. For this reason, Cpf1 nucleases have an advantage over the most widely used Cas9 nuclease systems that generate blunt-ended DSBs. With wider adoption and additional advances in reagent delivery, the Cpf1 genome editing platform has the potential to achieve even higher rates of targeted insertion, which would enable adoption of genome editing at all stages of a plant biotechnology program.

### LbCpf1 is also capable of generating insertion via HDR

In addition to FnCpf1, LbCpf1 was also screened for its ability to generate targeted integrations via HDR. Biolistic experiments were performed by using plasmids of LbCpf1 and respective crRNAs and repair templates (Tables S2 and S3) for *CAO1* target sites either #1 or #2 (GE0031 and GE0046, respectively, Table S3). Targeted integration events, as determined by PCR screening of callus tissues, were identified for both target sites at a frequency of 0% and 3%, respectively (Table 1). Sequencing of PCR amplicons of the upstream junction of events from GE0046 simply demonstrated an insertion that matches the design of the repair template (Fig. S11). These data demonstrate the ability of a second Cpf1 nuclease to generate targeted insertion via HDR in plants, albeit at a lower efficiency than was observed when mediated by FnCpf1.

### Indel detection in experiments with FnCpf1 and LbCpf1

**Figure 2:**
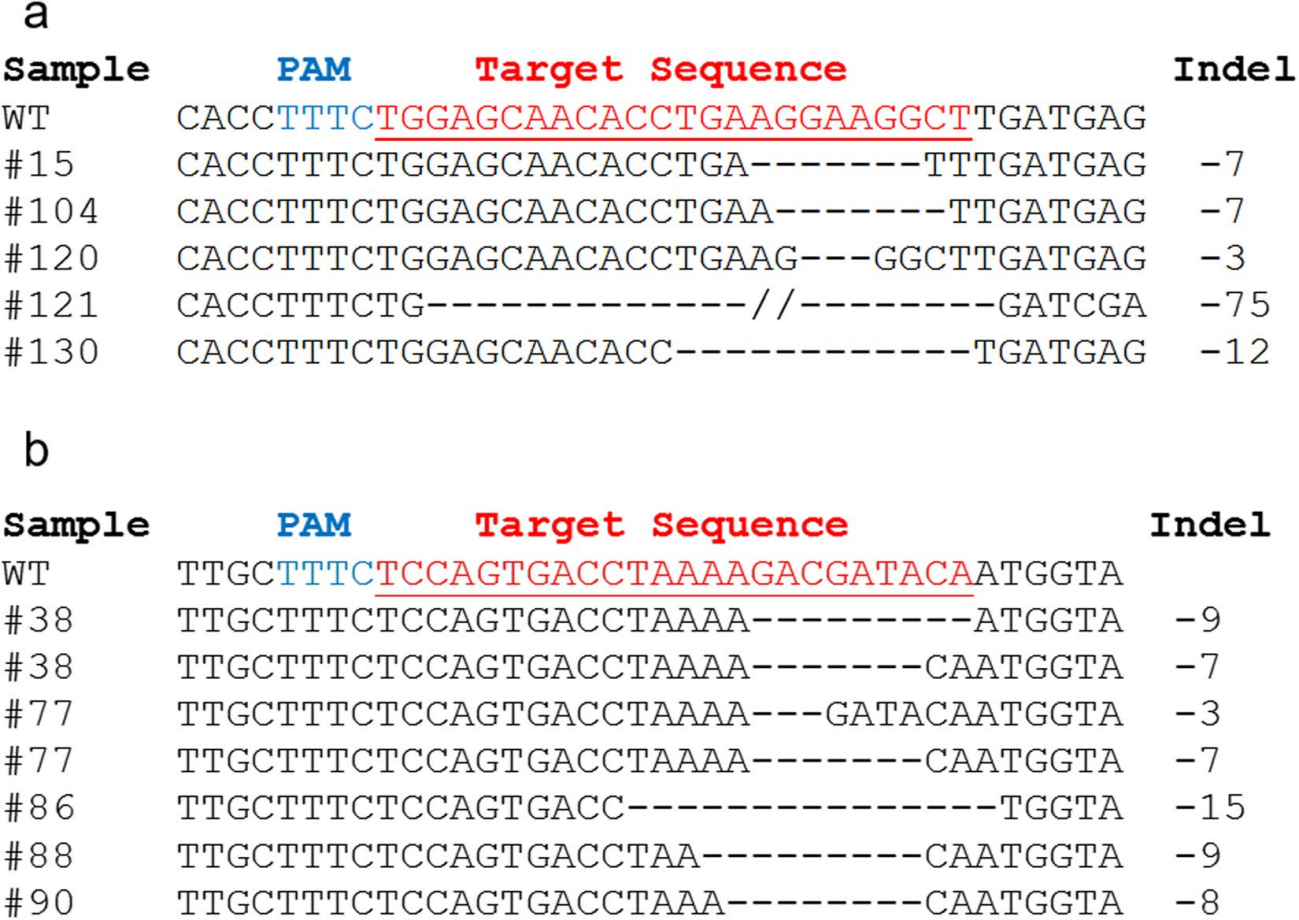
Identification of deletions generated by FnCpf1 and LbCpf1 in the *CAO1* gene. (**a**) Alignment of deletions identified from FnCpf1 experiments GE0001 and GE0001-R at *CAO1* site #1. (**b**) Alignment of indels identified from LbCpf1 experiment GE0046 at *CAO1* site #2.

In addition to screening callus from the previously presented experiments for targeted gene insertion, these callus fragments were also screened for the presence of indels at the target site. While these experiments were initially designed to generate HDR, there was the expectation that indels generated by NHEJ should be present as well. Calli from each experiment were screened using the T7 Endonuclease I (T7EI) assay (New England Biolabs)^27^. Indels were identified via T7EI at both sites within the *CAO1* gene with frequencies ranging from 2-32% (Table 1 and Figs S12 and S13). PCR products from callus with positive results from the T7EI assay were subcloned and sequenced (Fig. 2a and b). We found nucleotide deletions ranging from 3–75 bp (median 7 bp) for FnCpf1 and 3–52 bp (median 8 bp) for LbCpf1. It is also interesting to note that after sequencing multiple clones from single callus pieces, we found that callus from FnCpf1 bombardments appeared to have one predominant population of deletion mutants (Fig. 2a, sequence information from other clones with identical mutations are not shown), while samples from LbCpf1 tended to have multiple mutations in a single callus. For example, two clones from callus #77 (GE0046) produced nucleotide deletions of 3 and 7 bp, respectively (Fig. 2b). Multiple T0 plants were regenerated from experiment GE0046 (Table 1) calli that were shown to be positive for indels at the callus screening stage. The target region of these plants was amplified, subcloned, and sequenced (Fig. S14). One of the T0 plants (GE0046-40) is clearly chimeric, while the other two are hemizygous or bi-allelic.

The position of all identified indels are distal to the PAM site, which is consistent with the known enzymology of these nucleases^11^. The size and positioning of these mutations are similar to those recently published using Cpf1 nucleases in dicots and monocots^17, 18^. Our observations are also similar to those presented in mice and human cells^11, 16^. The observed frequencies of indel mutations in our biolistics experiments were lower than those observed with *Agrobacterium* mediated delivery of FnCpf1 and LbCpf1 in rice and tobacco^17, 18^. Clearly, optimization of component delivery has the potential to improve the frequency of edits. It is also interesting to note that FnCpf1, which has no to limited nuclease activity in human cells^11, 15^, performs especially well in plant systems, both in our experiments and other reports^17^. This again demonstrates the variability in the functionality (enzyme activity/stability) of genome editing nucleases across different host systems.

### Cpf1 nucleases are broadly applicable to plant genome editing

The results presented here demonstrate that both FnCpf1 and LbCpf1, when used together with crRNA and repairing template DNA, are capable of mediating both NHEJ and HDR based genome editing in plants. This is the first demonstration to our knowledge of Cpf1-mediated DNA insertion in a plant system. HDR in animal cells using Cpf1 nucleases has recently been shown in Zebrafish and *X. tropicalis* ^28^. These results and the results presented here are significant with respect to both advancement of Cpf1-based genome editing technology and plant biotechnology. The demonstration of Cpf1 nucleases to generate targeted insertion will expand the application of this genome editing platform across basic and applied science disciplines. The frequencies of targeted insertion demonstrated that the platform we established by using these nucleases, with the potential to increase with additional optimization of repairing template DNA and reagent delivery methods, etc., will enable wide-scale adoption of this technology in plant biotechnology from trait discovery to product development and trait stacking.

## Methods

### Plasmid construction

Cpf1 sequences were codon optimized for monocot plants and synthesized by GenScript. Cpf1 genes were subcloned via restriction digest cloning into expression vectors with a pUC19 backbone containing the 2 × 35S promoter and 3′UTR as well as a 35S terminator. Repair template plasmid was assembled by using the hot fusion cloning methodology^29^. Briefly, homology arms were amplified from rice genomic DNA and assembled with the hpt cassette driven by maize ubiquitin promoter and terminated by the 35S terminator in a pUC19 backbone. crRNA sequences were synthesized as G-Blocks Gene Fragments services from Integrated DNA technologies (IDT) and were cloned in between the rice U6 RNA promoter and terminator.

### Transformation

Biolistic-mediated transformation of rice embryogenic calli derived from mature seeds of *Oryza sativa* L. cv. Kitaake was performed as described in Chen *et al*.^30^, with slight modifications. DNA amounts of plasmids harboring Cpf1, crRNA and repairing template used in bombardment experiments were 0.5 µg, 0.5 µg and 1 µg per single shot respectively. The premixed DNA constructs were co-precipitated onto 0.6-µm gold microprojectiles as previously described^2^. Callus samples were bombarded once using PDS 1000/He biolistic system (BIO-RAD) at helium pressure of 1300 psi with a target distance of 6 cm and 27 in Hg vacuum in the chamber. Post-bombardment culture, hygromycin-B selection, and plant regeneration were performed as previously described^2^ with minor modifications.

### Mutation screening and DNA sequencing

DNA was extracted from rice callus or leaf tissue using the CTAB DNA extraction method. 100ng of purified genomic DNA was used for all PCR reactions. PCR assays for insertion screening were performed using GoTaq DNA polymerase (Promega) and the primers are listed in Table S1. Briefly, PCR products were electophoresed on 1% agarose gels before visualization. The PCR products were gel extracted and cloned into the pJET system (ThermoFisher). Plasmids sequencing (Sanger) service were provided by GenScript.

### T7EI screening

Target regions were amplified by using the PCR method described above. Cleaned up PCR products were treated with T7 Endonuclease I (New England Biolabs) in a 20 *µ*L reaction. Reactions were incubated at 37 °C for 15 min followed by the addition of 1.5 *µ*L 0.25 M EDTA to stop the reaction. Digests were electrophoresed on a 2% agarose gel prior to visualization. PCR products from calli positive for an indel analysis were cloned and sequenced as previously described.

## Electronic supplementary material

Supplementary Information
