# Supplementary material for: Precise insertion and guided editing of higher plant genomes using Cpf1 CRISPR nucleases
Source: Sci Rep. 2017 Sep 14;7:11606. doi: 10.1038/s41598-017-11760-6 (PMC5599503; doi:10.1038/s41598-017-11760-6)
Supplement: Supplementary file 1 — Supplementary Information [file 41598_2017_11760_MOESM1_ESM.pdf]

Title:

Precise insertion and guided editing of higher plant genomes using Cpf1 CRISPR nucleases

Author List:

Matthew B. Begemann[1,*], Benjamin N. Gray[1], Emma E. January[1], Gina C. Gordon[1], Yonghua He[1], Haijun Liu[1], Xingrong Wu[1], Thomas P. Brutnell[1,2], Todd C. Mockler[1,2], Mohammed Oufattole[1]

[1] Benson Hill Biosystems, 1100 Corporate Square Dr, St. Louis, MO 63132, USA

[2] Donald Danforth Plant Science Center, 975 N. Warson Road, St. Louis, MO 63132, USA

*Correspondence to mbegemann@bensonhillbio.com

Supplementary Information (SI)

SI tables:

Table S1: List of primers used in this study

Primer Name	Oligo Sequence (5'-3')	Purpose
OsCAO1 #1 Up Fwd (P1)	GGACAGCAGCAAGTGCCGAAAAATTC	CAO1 site #1 upstream junction screen
OsCAO1 #1 Up Rev (P2)	CCAAAATCCAGTACTAAAATCCAGATCCCCGA	CAO1 site #1 upstream junction screen
OsCAO1 #1 Dwn Fwd (P3)	GCTGCACTGCAGAAGCTTG	CAO1 site #1 downstream junction screen
OsCAO1 #1 Dwn Rev (P4)	TTGGAGCAAGGGAAGTGC	CAO1 site #1 downstream junction screen
OsCAO1 #1 Fwd	CCCCGCAAGTATTTGTATTCAC	PCR for T7EI assay on site #1
OsCAO1 #1 Rev	AAGAATCGCCAAACAACAACAAC	PCR for T7EI assay on site #1
OsCAO1 #2 Up Fwd	GGTTTCAAGGTTCTGTGATTCTTC	CAO1 site #2 upstream junction screen
OsCAO1 #2 Up Rev	CCAAAATCCAGTACTAAAATCCAGATCCCCGA	CAO1 site #2 upstream junction screen
OsCAO1 #2 Fwd	TGATAAGTTGGCACAAATGGAG	PCR for T7EI assay on site #2
OsCAO1 #2 Rev	ACTGAGCCAAGATGAAGAGGG	PCR for T7EI assay on site #2
FnCpf1 Fwd	GGCAAGTGGACTATCGCATC	Screen for presence of FnCpf1 gene
FnCpf1 Rev	CAGCTGGCACGACAGGTTTC	Screen for presence of FnCpf1 gene
hpt Rev	TCTCGATGAGCTGATGCTTTG	Screen for the presence of hpt gene

Table S2: List of plasmids used in this study

Plasmid ID	Description	Purpose
131272	35S::FnCpf1	Plant (monocot) codon optimized FnCpf1 expression vector
131277	35S::LbCpf1	Plant (monocot) codon optimized LbCpf1 expression vector
131608	U6::Target#1	FnCpf1 crRNA expression vector for CAO1 target #1
132033	U6::Target#1	LbCpf1 crRNA expression vector for CAO1 target #1
132054	U6::Target#2	LbCpf1 crRNA expression vector for CAO1 target #2
131760	ZmUbi::hpt	Repair template vector for CAO1 target #1
131632	ZmUbi::hpt	Repair template vector for CAO1 target #1 (PAM site mutated)
131633	ZmUbi::hpt	Repair template vector for CAO1 target #2

Table S3: List of plasmids for genome editing experiments

Genome Editing ID	Plasmid set for each experiment			Target site
	Cpf1	guide RNA	Repairing	
GE0001	131272	131608	131760	1
GE0001R	131272	131608	131632	1
GE0031	131277	132033	131632	1
GE0046	131277	132054	131633	2

SI figures:

S1: CAO1 site #1 target region

AGTATGTATTTTATATTAAAAATATTGCTATATTTTCTATAAATTTAATTAGACTGGACAAGTTTGACATGAATAAA
GTCAAAGCGGGTTATAAAATGAGGAAGTATCGTCCATTCCCTCGTTAAAAAAAGCAATTTGTAACTATAGTTATGAA
CGAGTACTGGTCATGTCTAGATTTTATTTATAGCCATTTCGTTTTGGGTCGAGAGCAGAGGGAATATCTTGCATGTA
AGTAAACATAGAGTTAAATCAAAGACATTTGTTCAGTTGTACCATATTCAACATACCTATAGCTTTGAAGCTACCGA
AATTCTTGTAGCGGACAGCAGCAAGTGCCGAAAAATTCTACTTCATCCGTTTCACAATGTAAGTCATTTTAGTATTT
TTCATATTCATATTAATGTTAATGAATCTAAATATATATATATATATGTCTAGATTCATTAACATTAATATGAATGT
GGGAAATGCGAATGACTTACATTTTAAAACGGAGGGAGTACCGCCAAACGTAATATTACCAGGTTGGAGGACCGATC
TGAAAAATATTCCGTGCAAGTACACATTGGACGTGGACCGCATTGCATACAAGTACAAGAGTTGAAACGCTTGGTTC
TCGAGGCTGCCACGTCAGAGCTTGAGCCTTCGAAGCGATCCCACGTGGCAGCACGTGGAAGGCTCGTGGAGATCGCG
GGCAGCTCCCCGCCTCTTATCGACAGGGCCACCTCGCCCGCGAAAATTATTATTTTTTTCGCCTTCCTTTAATAATA
CGCACGTATTTATACGATCTTACTTAAGCGAGCACCATTAGCATGCCACGTGTCACCGTAACAATCTACGTAGACCC
CGCAAGTATTTGTATTCACTAAACTTTCGTAAACGAAACTTGCAAAACACATCAAACCGGTCAAATTTGGACAGAAA
ATTCAGCCGAGATCGCAAATATTCGTTTCAAAACAAATTACTGTAACCGGCGACGCAGCCACGGGACGTGGATCGGA
CGGCGCGGAGGTGGACGGAGGAGGGATCAACGGCTGAGATGCGTTCCTGCGTGCCACGTGGTGCGCCGCGAGCAGAT
AAGGGTTCAGGGCGTCGCGACGGGGTCGAGAGATAAGGGGCGCGCGGCGAGTGGCGCGCCACGTCGCGTGGCCCAGA
TTGCGTTCTCCGGATTAGAGATTTCGAAATATCTTTTCGCGGAGAAAGAAAAAAAAATTAGACAAAACCACCCTCGG
TTGAGGAAAAATATGGGGAGTATTTGTGCGTTTTTATTTTTGTTTTTGGGTCGCACTGTTCGATCGTAGCATCCATG
ACCACTGTGGCATCGCTGTCTTTGCTGCCGCACTTGCTCATCA*Agcctt*CCTTCAGGTGTTGCTCCAGAAAGGTGAG
TTCTTCTTGTTGTTCCTTGAATCTGTTTTTGTTGTTGTTGTTTGGCGATTCTTGAATTTGTTTTGGGGTATCTGGCG
ATGGGAGGAACCATGTTTCTTGTTTGGTTTTTGGGTTCAGGTGGCCATTCTTGATGAAAAACTGAGTGTTTGAGTTT
GAGCAGTGCAATGGAGTTACCATTTTTGTTCTTCTGATTGGATTCTTTGTGATGGTTGATGTTTTTGTTCAGACAAT
GGTTTCAAGGTTCTGTGATTCTTCAGACCCCATATCTTAAAACCTGTTGTATTGAAGTAAGCAAAAAAACAAATCTT
GATCAAGGACAGCCTAGTTGCCAATTTTTCTTTGCAAATCTGAATGCAATTCAATCTCTTTCTTCCAGCAAATGCGT
GCAGCTTTCCCCCAGTAACCACAGGCTTATCTCTGACACTGATTTAACTAGATTTTGCTAATCTCTTTGATACTAGT
TTGTCTGCTAAAATAGAGTGCATGTGAGGTTGATGAAAATTGATGGTGACCTTGCTGATTGAAACTACACAGGGTGT
TGGTAGATATGGAGGAATCAAGGTGTATGCGGTGCTCGGTGATGATGGAGCTGACTATGCAAAGAACAACGCATGGG
AGGCCTTGTTCCATGTCGATGACCCGGGGCCAAGGGTTCCAATTGCAAAAGGCAAGTTCTTGGATGTCAACCAAGCT
CTTGAGGTGGTCCGGTTCGATATCCAGTATTGCGATTGGAGGGCGCGGCAGGACCTCCTCACCATCATGGTTCTTCA
CAACAAGGTAGGAAGCATTGGACAAGTCACAAGTTCAGAGAAGAGGTCAAAGCTTTCATAGTCTGAATTTTACAGAT
CATGGGATTCAAAATTGGACTGCATACTGAATAATGCTTGAGGTTGAAGTTTCGGATGACTGACATAGGTTAACTTA
AATGAATTTTTGAACATTGAAATGCAGGTGGTAGAGGTTCTTAATCCTTTAGCAAGGGAGTTCAAGTCAATTGGAAC
CTTGAGGAAAGAGCTTGCAGAATTACAGGAAGAATTGGCAAAAGCTCACAATCAGGTATTGTACTTTCAGGAGACAG
GAGCCAAATGAAAAACTTCAATATTATATGGATTCTGATGTTTTACATGTCTAATCCAGGTTCATCTGTCGGAAACT
AGAGTATCATCTGCCCTTGATAAGTTGGCACAAATGGAGACCCTTGTCAACGACAGACTGTTGCAAGATGGAGGCTC
TAGCGCATCTACAGCCGAGTGCACTTCCCTTGCTCCAAGCACGTCATCAGCGTCCCGTGTTGTAAACAAGAAACCTC
CTCGCCGGAGTCTGAACGTGTCTGGTCC

Figure S1: DNA sequence of target site #1 for the *O. sativa CAO1* locus (LOC_Os10g41780). The target site is shown in red highlight along with the PAM site in yellow highlight. The regions used for the ~1kb homology arms are highlighted in cyan. Primers binding sites used for the T7EI assay are shown in red (underlined). Italicized text indicates the 5-bp overhang generated by Cpf1-mediated DSB induction. Note the complementary DNA sequence of PAM site.

S2: crRNA Expression Cassette Design for CAO1 Site #1

TTTGTGAAAGTTGAATTACGGCATAGCCGAAGGAATAACAGAATCGTTTCACACTTTCGTAACAAAGGTCTTCTTA
TCATGTTTCAGACGATGGAGGCAAGGCTGATCAAAGTGATCAAGCACATAAACGCATTTTTTTACCATGTTTCACTC
CATAAGCGTCTGAGATTATCACAAGTCACGTCTAGTAGTTTGATGGTACACTAGTGACAATCAGTTCGTGCAGACA
GAGCTCATACTTGACTACTTGAGCGATTACAGGCGAAAGTGTGAAACGCATGTGATGTGGGCTGGGAGGAGGAG
AATATATACTAATGGGCCGTATCCTGATTTGGGCTGCGTCGGAAGGTGCAGCCCACGCGCGCCGTACCGCGCGGG
TGGCGCTGCTACCCACTTTAGTCCGTTGGATGGGGATCCGATGGTTTGCGCGGTGGCGTTGCGGGGGATGTTTAG
TACCACATCGGAAACCGAAAGACGATGGAACCAGCTTATAAACCCGCGCGCTGTAGTCAGCTTGAATTTCTACTGT
TGTAGATTGGAGCAACACCTGAAGGAAGGCTTTTTTTTGTTTT

Figure S2: The rice U6 promoter and terminator are shown in cyan and red, respectively. The mature crRNA hairpin is shown in purple and the target sequence for site #1 is shown in green.

S3: Predicted Marker Integration into CAO1 Site #1

AGTATGTATTTTATATTAAAAATATTGCTATATTTTCTATAAATTTAATTAGACTGGACAAGTTTGACAT
GAATAAAGTCAAAGCGGGTTATAAAATGAGGAAGTATCGTCCATTCCCTCGTTAAAAAAAGCAATTTGTA
ACTATAGTTATGAACGAGTACTGGTCATGTCTAGATTTTATTTATAGCCATTTCGTTTTGGGTCGAGAGC
AGAGGGAATATCTTGCATGTAAGTAAACATAGAGTTAAATCAAAGACATTTGTTCAGTTGTACCATATTC
AACATACCTATAGCTTTGAAGCTACCGAAATTCTTGTAGCGGACAGCAGCAAGTGCCGAAAAATTCTACT
TCATCCGTTTCACAATGTAAGTCATTTTAGTATTTTTCATATTCATATTAATGTTAATGAATCTAAATAT
ATATATATATATGTCTAGATTCATTAACATTAATATGAATGTGGGAAATGCGAATGACTTACATTTTAAA
ACGGAGGGAGTACCGCCAAACGTAATATTACCAGGTTGGAGGACCGATCTGAAAAATATTCCGTGCAAGT
ACACATTGGACGTGGACCGCATTGCATACAAGTACAAGAGTTGAAACGCTTGGTTCTCGAGGCTGCCACG
TCAGAGCTTGAGCCTTCGAAGCGATCCCACGTGGCAGCACGTGGAAGGCTCGTGGAGATCGCGGGCAGCT
CCCCGCCTCTTATCGACAGGGCCACCTCGCCCGCGAAAATTATTATTTTTTTCGCCTTCCTTTAATAATA
CGCACGTATTTATACGATCTTACTTAAGCGAGCACCATTAGCATGCCACGTGTCACCGTAACAATCTACG
TAGACCCCGCAAGTATTTGTATTCACTAAACTTTCGTAAACGAAACTTGCAAAACACATCAAACCGGTCA
AATTTGGACAGAAAATTCAGCCGAGATCGCAAATATTCGTTTCAAAACAAATTACTGTAACCGGCGACGC
AGCCACGGGACGTGGATCGGACGGCGCGGAGGTGGACGGAGGAGGGATCAACGGCTGAGATGCGTTCCTG
CGTGCCACGTGGTGCGCCGCGAGCAGATAAGGGTTCAGGGCGTCGCGACGGGGTCGAGAGATAAGGGGCG
CGCGGCGAGTGGCGCGCCACGTCGCGTGGCCCAGATTGCGTTCTCCGGATTAGAGATTTCGAAATATCTT
TTCGCGGAGAAAGAAAAAAAAATTAGACAAAACCACCCTCGGTTGAGGAAAAATATGGGGAGTATTTGTG
CGTTTTTATTTTTGTTTTTGGGTCGCACTGTTCGATCGTAGCATCCATGACCACTGTGGCATCGCTGTCT
TTGCTGCCGCACTTGCTCATCAAagctgaattaacgccgaattaattcgggggatctggattttagtact
ggattttggttttaggaattagaaattttattgatagaagtattttacaaatacaaatacatactaaggg
tttcttatatgctcaacacatgagcgaaaccctataggaaccctaattcccttatctgggaactactcac
acattattatggagaaactcgagcttgtcgatcgacagatcccggtcggcatctactctatttctttgcc
ctcggacgagtgctggggcgtcggtttccactatcggcgagtacttctacacagccatcggtccagacgg
ccgcgcttctgcgggcgatttgtgtacgcccgacagtcccggctccggatcggacgattgcgtcgcatcg
accctgcgcccaagctgcatcatcgaaattgccgtcaaccaagctctgatagagttggtcaagaccaatg
cggagcatatacgcccggagtcgtggcgatcctgcaagctccggatgcctccgctcgaagtagcgcgtct
gctgctccatacaagccaaccacggcctccagaagaagatgttggcgacctcgtattgggaatccccgaa
catcgcctcgctccagtcaatgaccgctgttatgcggccattgtccgtcaggacattgttggagccgaaa
tccgcgtgcacgaggtgccggacttcggggcagtcctcggcccaaagcatcagctcatcgagagcctgcg
cgacggacgcactgacggtgtcgtccatcacagtttgccagtgatacacatggggatcagcaatcgcgca
tatgaaatcacgccatgtagtgtattgaccgattccttgcggtccgaatgggccgaacccgctcgtctgg
ctaagatcggccgcagcgatcgcatccatagcctccgcgaccggttgtagaacagcgggcagttcggttt
caggcaggtcttgcaacgtgacaccctgtgaacggcgggagatgcaataggtcaggctctcgctaaactc

cccaatgtcaagcacttccggaatcgggagcgcggccgatgcaaagtgccgataaacataacgatctttg
tagaaaccatcggcgcagctatttacccgcaggacatatccacgccctcctacatcgaagctgaaagcac
gagattcttcgccctccgagagctgcatcaggtcggagacgctgtcgaacttttcgatcagaaacttctc
gacagacgtcgcggtgagttcaggctttttcatatctcattgcccgggaagcttatcgtctacctgcaga
agtaacaccaaacaacagggtgagcatcgacaaaagaaacagtaccaagcaaataaatagcgtatgaagg
cagggctaaaaaaatccacatatagctgctgcatatgccatcatccaagtatatcaagatcaaaataatt
ataaaacatacttgtttattataatagataggtactcaaggttagagcatatgaatagatgctgcatatg
ccatcatgtatatgcatcagtaaaacccacatcaacatgtatacctatcctagatcgatatttccatcca
tcttaaactcgtaactatgaagatgtatgacacacacatacagttccaaaattaataaatacaccaggta
gtttgaaacagtattctactccgatctagaacgaatgaacgaccgcccaaccacaccacatcatcacaac
caagcgaacaaaaagcatctctgtatatgcatcagtaaaacccgcatcaacatgtatacctatcctagat
cgatatttccatccatcatcttcaattcgtaactatgaatatgtatggcacacacatacagatccaaaat
taataaatccaccaggtagtttgaaacagaattctactccgatctagaacgaccgcccaaccagaccaca
tcatcacaaccaagacaaaaaaaagcatgaaaagatgacccgacaaacaagtgcacggcatatattgaaa
taaaggaaaagggcaaaccaaaccctatgcaacgaaacaaaaaaaatcatgaaatcgatcccgtctgcgg
aacggctagagccatcccaggattccccaaagagaaacactggcaagttagcaatcagaacgtgtctgac
gtacaggtcgcatccgtgtacgaacgctagcagcacggatctaacacaaacacggatctaacacaaacat
gaacagaagtagaactaccgggccctaaccatggaccggaacgccgatctagagaaggtagagaggggg
gggggggaggacgagcggcgtaccttgaagcggaggtgccgacgggtggatttgggggagatctggttgt
gtgtgtgtgcgctccgaacaacacgaggttggggaaagagggtgtggagggggtgtctatttattacggc
gggcgaggaagggaaagcgaaggagcggtgggaaaggaatcccccgtagctgccgtgccgtgagaggagg
aggaggccgcctgccgtgccggctcacgtctgccgctccgccacgcaatttctggatgccgacagcggag
caagtccaacggtggagcggaactctcgagaggggtccagaggcagcgacagagatgccgtgccgtctgc
ttcgcttggcccgacgcgacgctgctggttcgctggttggtgtccgttagactcgtcgacggcgtttaac
aggctggcattatctactcgaaacaagaaaaatgtttccttagtttttttaatttcttaaagggtatttg
tttaatttttagtcactttattttattctattttatatctaaattattaaataaaaaaactaaaatagag
ttttagttttcttaatttagaggctaaaatagaataaaatagatgtactaaaaaaattagtctataaaaa
ccattaaccctaaaccctaaatggatgtactaataaaatggatgaagtattatataggtgaagctatttg
caaaaaaaaaggagaacacatgcacactaaaaagataaaactgtagagtcctgttgtcaaaatactcaat
tgtcctttagaccatgtctaactgttcatttatatgattctctaaaacactgatattattgtagtagtat
agattatattattcgtagagtaaagtttaaatatatgtataaagatagataaactgcacttcaaacaagt
gtgacaaaaaaaatatgtggtaatttttataacttagacatgcaatgctcattatctctagagagggggc
acgaccgggtcacgctgcactgcagaagcttgctgCCTTCAGGTGTTGCTCCAGAAAGGTGAGTTCTTCT
TGTTGTTCCTTGAATCTGTTTTTGTTGTTGTTGTTTGGCGATTCTTGAATTTGTTTTGGGGTATCTGGCG
ATGGGAGGAACCATGTTTCTTGTTTGGTTTTTGGGTTCAGGTGGCCATTCTTGATGAAAAACTGAGTGTT
TGAGTTTGAGCAGTGCAATGGAGTTACCATTTTTGTTCTTCTGATTGGATTCTTTGTGATGGTTGATGTT
TTTGTTCAGACAATGGTTTCAAGGTTCTGTGATTCTTCAGACCCCATATCTTAAAACCTGTTGTATTGAA
GTAAGCAAAAAAACAAATCTTGATCAAGGACAGCCTAGTTGCCAATTTTTCTTTGCAAATCTGAATGCAA
TTCAATCTCTTTCTTCCAGCAAATGCGTGCAGCTTTCCCCCAGTAACCACAGGCTTATCTCTGACACTGA
TTTAACTAGATTTTGCTAATCTCTTTGATACTAGTTTGTCTGCTAAAATAGAGTGCATGTGAGGTTGATG
AAAATTGATGGTGACCTTGCTGATTGAAACTACACAGGGTGTTGGTAGATATGGAGGAATCAAGGTGTAT
GCGGTGCTCGGTGATGATGGAGCTGACTATGCAAAGAACAACGCATGGGAGGCCTTGTTCCATGTCGATG
ACCCGGGGCCAAGGGTTCCAATTGCAAAAGGCAAGTTCTTGGATGTCAACCAAGCTCTTGAGGTGGTCCG
GTTCGATATCCAGTATTGCGATTGGAGGGCGCGGCAGGACCTCCTCACCATCATGGTTCTTCACAACAAG
GTAGGAAGCATTGGACAAGTCACAAGTTCAGAGAAGAGGTCAAAGCTTTCATAGTCTGAATTTTACAGAT
CATGGGATTCAAAATTGGACTGCATACTGAATAATGCTTGAGGTTGAAGTTTCGGATGACTGACATAGGT
TAACTTAAATGAATTTTTGAACATTGAAATGCAGGTGGTAGAGGTTCTTAATCCTTTAGCAAGGGAGTTC
AAGTCAATTGGAACCTTGAGGAAAGAGCTTGCAGAATTACAGGAAGAATTGGCAAAAGCTCACAATCAGG

TATTGTACTTTCAGGAGACAGGAGCCAAATGAAAAACTTCAATATTATATGGATTCTGATGTTTTACATG
TCTAATCCAGGTTCATCTGTCGGAAACTAGAGTATCATCTGCCCTTGATAAGTTGGCACAAATGGAGACC
CTTGTCAACGACAGACTGTTGCAAGATGGAGGCTCTAGCGCATCTACAGCCGAGTGCACTTCCCTTGCTC
CAAGCACGTCATCAGCGTCCCGTGTTGTAAACAAGAAACCTCCTCGCCGGAGTCTGAACGTGTCTGGTCC

Figure S3: Sequence of targeted integration of hygromycin-resistance marker into CAO1 site #1. Homology arms used in the repair template are shown in cyan. The insertion is comprised of the ZmUbi1 promoter (green), hpt hygromycin phosphotransferase gene (yellow), and CaMV 35S terminator (purple). Primer binding sites used in screening for integrations are shown in red underline, corresponding to P1, P2, P3, and P4.

S4: CAO1 site #2 target region

TGGCGATTCTTGAATTTGTTTTGGGGTATCTGGCGATGGGAGGAACCATGTTTCTTGTTTGGTTTTTGGG
TTCAGGTGGCCATTCTTGATGAAAAACTGAGTGTTTGAGTTTGAGCAGTGCAATGGAGTTACCATTTTTG
TTCTTCTGATTGGATTCTTTGTGATGGTTGATGTTTTTGTTCAGACAATGGTTTCAAGGTTCTGTGATTC
TTCAGACCCCATATCTTAAAACCTGTTGTATTGAAGTAAGCAAAAAAACAAATCTTGATCAAGGACAGCC
TAGTTGCCAATTTTTCTTTGCAAATCTGAATGCAATTCAATCTCTTTCTTCCAGCAAATGCGTGCAGCTT
TCCCCCAGTAACCACAGGCTTATCTCTGACACTGATTTAACTAGATTTTGCTAATCTCTTTGATACTAGT
TTGTCTGCTAAAATAGAGTGCATGTGAGGTTGATGAAAATTGATGGTGACCTTGCTGATTGAAACTACAC
AGGGTGTTGGTAGATATGGAGGAATCAAGGTGTATGCGGTGCTCGGTGATGATGGAGCTGACTATGCAAA
GAACAACGCATGGGAGGCCTTGTTCCATGTCGATGACCCGGGGCCAAGGGTTCCAATTGCAAAAGGCAAG
TTCTTGGATGTCAACCAAGCTCTTGAGGTGGTCCGGTTCGATATCCAGTATTGCGATTGGAGGGCGCGGC
AGGACCTCCTCACCATCATGGTTCTTCACAACAAGGTAGGAAGCATTGGACAAGTCACAAGTTCAGAGAA
GAGGTCAAAGCTTTCATAGTCTGAATTTTACAGATCATGGGATTCAAAATTGGACTGCATACTGAATAAT
GCTTGAGGTTGAAGTTTCGGATGACTGACATAGGTTAACTTAAATGAATTTTTGAACATTGAAATGCAGG
TGGTAGAGGTTCTTAATCCTTTAGCAAGGGAGTTCAAGTCAATTGGAACCTTGAGGAAAGAGCTTGCAGA
ATTACAGGAAGAATTGGCAAAAGCTCACAATCAGGTATTGTACTTTCAGGAGACAGGAGCCAAATGAAAA
ACTTCAATATTATATGGATTCTGATGTTTTACATGTCTAATCCAGGTTCATCTGTCGGAAACTAGAGTAT
CATCTGCCCTTGATAAGTTGGCACAAATGGAGACCCTTGTCAACGACAGACTGTTGCAAGATGGAGGCTC
TAGCGCATCTACAGCCGAGTGCACTTCCCTTGCTCCAAGCACGTCATCAGCGTCCCGTGTTGTAAACAAG
AAACCTCCTCGCCGGAGTCTGAACGTGTCTGGTCCAGTGCAGCCATACAATCCCAGTCTGAAGAACTTCT
GGTACCCAGTTGCTTTCTCCAGTGACCTAAAAGAC*GATACA*ATGGTAAAAACAACGTGCGATTCAGGCAT
TTTTTTACGAGGTCAGAGTTAGCAGTTGCAGAAACTTATGTTTATTTGATGTGGTTTGCGCATTCTCAGG
TGCCAATAGATTGTTTTGAGGAGCAGTGGGTAATTTTCCGAGGAAAGGATGGGAGACCTGGATGTGTTAT
GAACACATGTGCTCACAGAGCTTGCCCTCTTCATCTTGGCTCAGTTAATGAGGGCAGAATCCAATGCCCT
TACCATGGTAAGAAAAGACAGCTTTACATGAACTTTCATTTCTGCATGCTTCCGCATTTATTTCTGAAGT
CTTCAGATGAAAATTTGCCAACAGAGAGAAGTTGCGAAAGTAGTATTTCAGTTTTTTTTTGTTTTCATAA
CCTTCAGGTTGGGAGTATTCAACTGATGGAAAATGTGAGAAAATGCCATCCACAAAGATGCTCAACGTGC
GCATCCGGTCATTACCATGCTTTGAGCAAGAAGGAATGGTTTGGATATGGCCTGGCAATGACCCACCGAA
GTCGACTATCCCTTCTCTGCTGCCTCCTTCAGGATTTACAATTCACGCAGAGGTAAAAGGAGATCATGTC
ATGCTGCAGCAACCATACTATGTGGAACTGTCCTGTGCATTTCAAGATTTTTACTGGACTGAAAAGTAAT
GGAATTGTTCTTGATCATCAACAGATAGTGATGGAGCTACCAGTGGAGCATGGACTTCTTCTGGACAATC
TATTAGATCTTGCTCATGCTCCTTTTACTCATACATCCACCTTTGCCAAGGGTTGGAGTGTTCCAAGGTA
TTTACATACATATATTTTCACAGCCATGTGGAATTCTTTTTTTTTTTTTTAGAAAATGGATAGCCATGTG
GAAATGTCTTCAGAAATCATCATGCTGATGCTATGTTCATTTCCCAGCTTGGTGAAGTTCTTGACACCTT

CATCTGGGCTTCAAGGATACTGGGATCCATACCCGATCGACATGGAATTTCGACCACCATGCATGGTGTT
GTCAACCATTGGCATCTCAAAGCCTGGAAAACTAGAGGGGAAGAGCACCAAGCAATGTTCGACGCATCTC
CACCAGCTCCATATCTGTTTGCCCTCCTCTAGGAATAAAACCAGGCTGCTCTACCGGATGTCTCTCGACT
TCGCTCCATGGATCAAGCATGTCCCTTTCATGCATATACTATGGTCACATTTTGCTGAGAAGGTGAGTCC
GAAAAATTCAGAGCAACTTCATACTAAACTGTTGTCCATCTCATGTCATTACAGTTTGCACCTGACTATA
GTATGCGGTTACCTTTGTTTTGCATACTGTCCTCTATACAACATCTATAATAATCTTGTATGATCTTTCT
GCAGGTCTTGAATGAGGATCTTCGACTCGTGCTCGGGCAGCAAGAACGGATGATCAATGGCGCAAATGTC
TGGAACTGGCCAGTATCATATGACAAGCTTGGTATCCGGTATCGGTTGTGGAGAGACGCCATTGAGAGGG

Figure S4: Sequence of target site #2 for the *O. sativa* CAO1 locus (LOC_Os10g41780). The target site is shown in red highlight along with the PAM site in yellow highlight. The regions used for the ~1kb homology arms are highlighted in cyan. Primers used for the T7EI assay are shown in red underline. Italicized text indicates the 5-bp overhang generated by Cpf1-mediated DSB induction.

S5: crRNA Expression Cassette Design for CAO1 Site #2

TTTGTGAAAGTTGAATTACGGCATAGCCGAAGGAATAACAGAATCGTTTCACACTTTCGTAACAAAGGTCTTCTTA
TCATGTTTCAGACGATGGAGGCAAGGCTGATCAAAGTGATCAAGCACATAAACGCATTTTTTTACCATGTTTCACTC
CATAAGCGTCTGAGATTATCACAAGTCACGTCTAGTAGTTTGATGGTACACTAGTGACAATCAGTTCGTGCAGACA
GAGCTCATACTTGACTACTTGAGCGATTACAGGCGAAAGTGTGAAACGCATGTGATGTGGGCTGGGAGGAGGAG
AATATATACTAATGGGCCGTATCCTGATTTGGGCTGCGTCGGAAGGTGCAGCCCACGCGCGCCGTACCGCGCGGG
TGGCGCTGCTACCCACTTTAGTCCGTTGGATGGGGATCCGATGGTTTGCGCGGTGGCGTTGCGGGGGATGTTTAG
TACCACATCGGAAACCGAAAGACGATGGAACCAGCTTATAAACCCGCGCGCTGTAGTCAGCTTGAATTTCTACTGT
TGTAGATTCCAGTGACCTAAAAGACGATACATTTTTTTGTTTT

Figure S5: The rice U6 promoter and terminator are shown in cyan and red, respectively. The mature crRNA hairpin is shown in purple and the target sequence for site #2 is shown in green.

S6: Predicted Marker Integration into CAO1 Site #2

CCTTCAGGTGTTGCTCCAGAAAGGTGAGTTCTTCTTGTTGTTCCTTGAATCTGTTTTTGTTGTTGTTGTT
TGGCGATTCTTGAATTTGTTTTGGGGTATCTGGCGATGGGAGGAACCATGTTTCTTGTTTGGTTTTTGGG
TTCAGGTGGCCATTCTTGATGAAAAACTGAGTGTTTGAGTTTGAGCAGTGCAATGGAGTTACCATTTTTG
TTCTTCTGATTGGATTCTTTGTGATGGTTGATGTTTTTGTTCAGACAATGGTTTCAAGGTTCTGTGATTC
TTCAGACCCCATATCTTAAAACCTGTTGTATTGAAGTAAGCAAAAAAACAAATCTTGATCAAGGACAGCC
TAGTTGCCAATTTTTCTTTGCAAATCTGAATGCAATTCAATCTCTTTCTTCCAGCAAATGCGTGCAGCTT
TCCCCCAGTAACCACAGGCTTATCTCTGACACTGATTTAACTAGATTTTGCTAATCTCTTTGATACTAGT
TTGTCTGCTAAAATAGAGTGCATGTGAGGTTGATGAAAATTGATGGTGACCTTGCTGATTGAAACTACAC
AGGGTGTTGGTAGATATGGAGGAATCAAGGTGTATGCGGTGCTCGGTGATGATGGAGCTGACTATGCAAA
GAACAACGCATGGGAGGCCTTGTTCCATGTCGATGACCCGGGGCCAAGGGTTCCAATTGCAAAAGGCAAG
TTCTTGGATGTCAACCAAGCTCTTGAGGTGGTCCGGTTCGATATCCAGTATTGCGATTGGAGGGCGCGGC
AGGACCTCCTCACCATCATGGTTCTTCACAACAAGGTAGGAAGCATTGGACAAGTCACAAGTTCAGAGAA
GAGGTCAAAGCTTTCATAGTCTGAATTTTACAGATCATGGGATTCAAAATTGGACTGCATACTGAATAAT
GCTTGAGGTTGAAGTTTCGGATGACTGACATAGGTTAACTTAAATGAATTTTTGAACATTGAAATGCAGG
TGGTAGAGGTTCTTAATCCTTTAGCAAGGGAGTTCAAGTCAATTGGAACCTTGAGGAAAGAGCTTGCAGA
ATTACAGGAAGAATTGGCAAAAGCTCACAATCAGGTATTGTACTTTCAGGAGACAGGAGCCAAATGAAAA
ACTTCAATATTATATGGATTCTGATGTTTTACATGTCTAATCCAGGTTCATCTGTCGGAAACTAGAGTAT
CATCTGCCCTTGATAAGTTGGCACAAATGGAGACCCTTGTCAACGACAGACTGTTGCAAGATGGAGGCTC
TAGCGCATCTACAGCCGAGTGCACTTCCCTTGCTCCAAGCACGTCATCAGCGTCCCGTGTTGTAAACAAG
AAACCTCCTCGCCGGAGTCTGAACGTGTCTGGTCCAGTGCAGCCATACAATCCCAGTCTGAAGAACTTCT
GGTACCCAGTTGCTTTCTCCAGTGACCTAAAAGACagctgaattaacgccgaattaattcggggatctg
gattttagtactggattttggttttaggaattagaaattttattgatagaagtattttacaaatacaaat
acatactaagggtttcttatatgctcaacacatgagcgaaaccctataggaaccctaattcccttatctg
ggaactactcacacattattatggagaaactcgagcttgtcgatcgacagatcccggtcggcatctactc
tatttctttgccctcggacgagtgctggggcgtcggtttccactatcggcgagtacttctacacagccat
cggtccagacggccgcgcttctgcgggcgatttgtgtacgcccgacagtcccggctccggatcggacgat
tgcgtcgcatcgaccctgcgcccaagctgcatcatcgaaattgccgtcaaccaagctctgatagagttgg
tcaagaccaatgcggagcatatacgcccggagtcgtggcgatcctgcaagctccggatgcctccgctcga
agtagcgcgtctgctgctccatacaagccaaccacggcctccagaagaagatgttggcgacctcgtattg
ggaatccccgaacatcgcctcgctccagtcaatgaccgctgttatgcggccattgtccgtcaggacattg
ttggagccgaaatccgcgtgcacgaggtgccggacttcggggcagtcctcggcccaaagcatcagctcat
cgagagcctgcgcgacggacgcactgacggtgtcgtccatcacagtttgccagtgatacacatggggatc

agcaatcgcgcatatgaaatcacgccatgtagtgtattgaccgattccttgcggtccgaatgggccgaac
ccgctcgtctggctaagatcggccgcagcgatcgcatccatagcctccgcgaccggttgtagaacagcgg
gcagttcggtttcaggcaggtcttgcaacgtgacaccctgtgaacggcgggagatgcaataggtcaggct
ctcgctaaactccccaatgtcaagcacttccggaatcgggagcgcggccgatgcaaagtgccgataaaca
taacgatctttgtagaaaccatcggcgcagctatttacccgcaggacatatccacgccctcctacatcga
agctgaaagcacgagattcttcgccctccgagagctgcatcaggtcggagacgctgtcgaacttttcgat
cagaaacttctcgacagacgtcgcggtgagttcaggcttttttcatatctcattgcccgggaagcttatcg
tctacctgcagaagtaacaccaaacaacagggtgagcatcgacaaaagaaacagtaccaagcaaataaat
agcgtatgaaggcagggctaaaaaaatccacatatagctgctgcatatgccatcatccaagtatatcaag
atcaaaataattataaaacatacttgtttattataatagataggtactcaaggttagagcatatgaatag
atgctgcatatgccatcatgtatatgcatcagtaaaacccacatcaacatgtatacctatcctagatcga
tatttccatccatcttaaactcgtaactatgaagatgtatgacacacacatacagttccaaaattaataa
atacaccaggtagtttgaaacagtattctactccgatctagaacgaatgaacgaccgcccaaccacacca
catcatcacaaccaagcgaacaaaaagcatctctgtatatgcatcagtaaaacccgcatcaacatgtata
cctatcctagatcgatatttccatccatcatcttcaattcgtaactatgaatatgtatggcacacacata
cagatccaaaattaataaatccaccaggtagtttgaaacagaattctactccgatctagaacgaccgccc
aaccagaccacatcatcacaaccaagacaaaaaaaagcatgaaaagatgacccgacaaacaagtgcacgg
catatattgaaataaaggaaaagggcaaaccaaaccctatgcaacgaaacaaaaaaaatcatgaaatcga
tcccgtctgcggaacggctagagccatcccaggattccccaaagagaaacactggcaagttagcaatcag
aacgtgtctgacgtacaggtcgcatccgtgtacgaacgctagcagcacggatctaacacaaacacggatc
taacacaaacatgaacagaagtagaactaccgggccctaaccatggaccggaacgccgatctagagaagg
tagagaggggggggggggaggacgagcggcgtaccttgaagcggaggtgccgacgggtggatttggggg
agatctggttgtgtgtgtgtgcgctccgaacaacacgaggttggggaaagagggtgtggaggggggtgtct
atttattacggcgggcgaggaagggaaagcgaaggagcggtgggaaaggaatcccccgtagctgccgtgc
cgtgagaggaggaggaggccgcctgccgtgccggctcacgtctgccgctccgccacgcaatttctggatg
ccgacagcggagcaagtccaacggtggagcggaactctcgagaggggtccagaggcagcgacagagatgc
cgtgccgtctgcttcgcttggcccgacgcgacgctgctggttcgctggttggtgtccgttagactcgtcg
acggcgtttaacaggctggcattatctactcgaaacaagaaaaatgtttccttagtttttttaatttctt
aaagggtatttgtttaattttagtcactttattttattctattttatatctaaattattaaataaaaaa
actaaaatagagttttagttttcttaatttagaggctaaaatagaataaaatagatgtactaaaaaaatt
agtctataaaaaccattaaccctaaaccctaaatggatgtactaataaaatggatgaagtattatatagg
tgaagctatttgcaaaaaaaaaggagaacacatgcacactaaaaagataaaactgtagagtcctgttgtc
aaaatactcaattgtcctttagaccatgtctaactgttcatttatatgattctctaaaacactgatatta
ttgtagtagtatagattatattattcgtagagtaaagtttaaatatatgtataaagatagataaactgca
cttcaaacaagtgtgacaaaaaaaatatgtggtaattttttataacttagacatgcaatgctcattatct
ctagagaggggcacgaccgggtcacgctgcactgcagaagcttgctgAATGGTAAAAACAACGTGCGATT
CAGGCATTTTTTTACGAGGTCAGAGTTAGCAGTTGCAGAAACTTATGTTTATTTGATGTGGTTTGCGCAT
TCTCAGGTGCCAATAGATTGTTTTGAGGAGCAGTGGGTAATTTTCCGAGGAAAGGATGGGAGACCTGGAT
GTGTTATGAACACATGTGCTCACAGAGCTTGCCCTCTTCATCTTGGCTCAGTTAATGAGGGCAGAATCCA
ATGCCCTTACCATGGTAAGAAAAGACAGCTTTACATGAACTTTCATTTCTGCATGCTTCCGCATTTATTT
CTGAAGTCTTCAGATGAAAATTTGCCAACAGAGAGAAGTTGCGAAAGTAGTATTTCAGTTTTTTTTTGTT
TTCATAACCTTCAGGTTGGGAGTATTCAACTGATGGAAAATGTGAGAAAATGCCATCCACAAAGATGCTC
AACGTGCGCATCCGGTCATTACCATGCTTTGAGCAAGAAGGAATGGTTTGGATATGGCCTGGCAATGACC
CACCGAAGTCGACTATCCCTTCTCTGCTGCCTCCTTCAGGATTTACAATTCACGCAGAGGTAAAAGGAGA
TCATGTCATGCTGCAGCAACCATACTATGTGGAACTGTCCTGTGCATTTCAAGATTTTTACTGGACTGAA
AAGTAATGGAATTGTTCTTGATCATCAACAGATAGTGATGGAGCTACCAGTGGAGCATGGACTTCTTCTG
GACAATCTATTAGATCTTGCTCATGCTCCTTTTACTCATACATCCACCTTTGCCAAGGGTTGGAGTGTTC
CAAGGTATTTACATACATATATTTCACAGCCATGTGGAATTCTTTTTTTTTTTTTTAGAAAATGGATAG

CCATGTGGAAATGTCTTCAGAAATCATCATGCTGATGCTATGTTCATTTCCCAGCTTGGTGAAGTTCTTG
ACACCTTCATCTGGGCTTCAAGGATACTGGGATCCATACCCGATCGACATGGAATTTCGACCACCATGCA
TGGTGTTGTCAACCATTGGCATCTCAAAGCCTGGAAAACTAGAGGGGAAGAGCACCAAGCAATGTTCGAC
GCATCTCCACCAGCTCCATATCTGTTTGCCCTCCTCTAGGAATAAAACCAGGCTGCTCTACCGGATGTCT
CTCGACTTCGCTCCATGGATCAAGCATGTCCCTTTCATGCATATACTATGGTCACATTTTGCTGAGAAGG
TGAGTCCGAAAAATTCAGAGCAACTTCATACTAAACTGTTGTCCATCTCATGTCATTACAGTTTGCACCT
GACTATAGTATGCGGTTACCTTTGTTTTGCATACTGTCCTCTATACAACATCTATAATAATCTTGTATGA

Figure S6: Sequence of targeted integration of hygromycin resistance marker into CAO1 site #2. Homology arms used in the repair template are shown in cyan. The insertion is comprised of the ZmUbi1 promoter (green), hpt hygromycin phosphotransferase gene (yellow), and CaMV 35S terminator (purple). Primer binding sites used in screening for integrations are shown in red underline.

S7: PCR of calli from GE0001

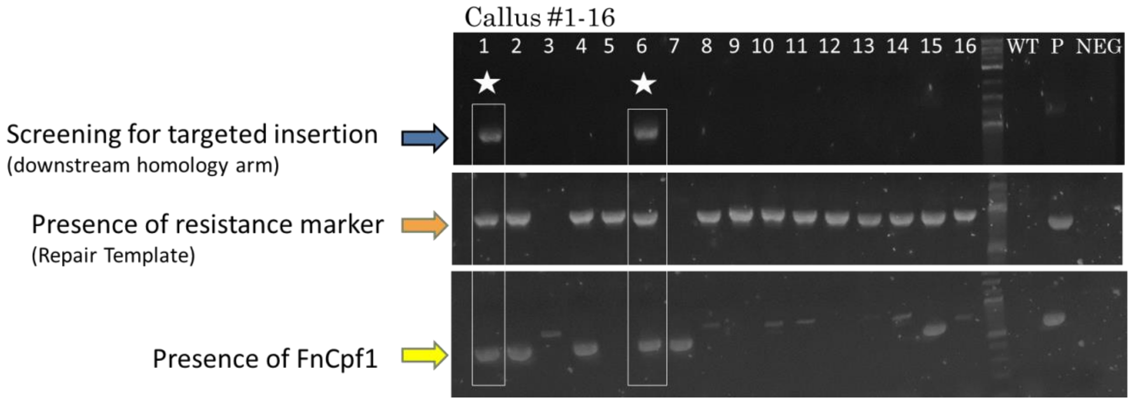

Figure S7: Representative PCR results of initial set of callus lines from GE0001 (callus #21 is not shown). All PCR experiments contain a wildtype (WT) control, plasmid (P) control, and no DNA (NEG for negative) control. Panel 1 is a PCR screening using a primer (P3) located in the ZmUbi promoter region of the hpt resistance marker and a primer (P4) in the genomic region downstream of the homology arm (P4). The second panel is a PCR screen using primers OsCAO1 #1 Fwd and hpt Rev (specific to the hpt gene, Table S1). The third panel is a PCR screening using primers specific to the FnCpf1 gene (FnCpf1 Fwd and FnCpf1 Rev). Some lanes for this PCR assay have a non-specific band that does not match the plasmid control band.

S8: Alignment of Sequencing Results from GE0001 Calli Positive for Insertions

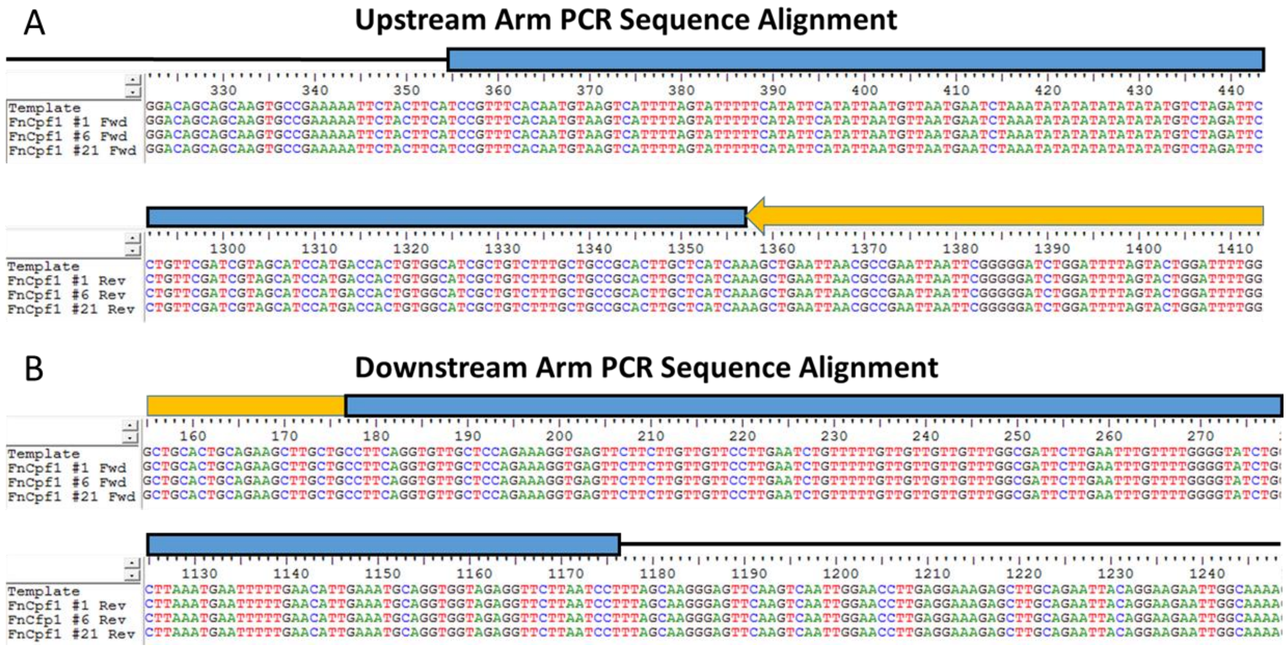

Figure S8: Alignments of sequencing results from two separate PCR amplicons for the insertion junction regions for callus #1, #6, and #21. Genomic sequence is shown as a black line, homology arm shown as blue rectangle, and the hpt marker is shown as a yellow arrow. (A) Alignments of the genomic region-homology arm and homology arm-marker from the

upstream PCR amplicon. (B) Alignments of the marker-homology arm and homology arm-genomic region from the downstream PCR amplicon.

S9: PCR Confirmation of Plants Derived from GE0001 Callus #6

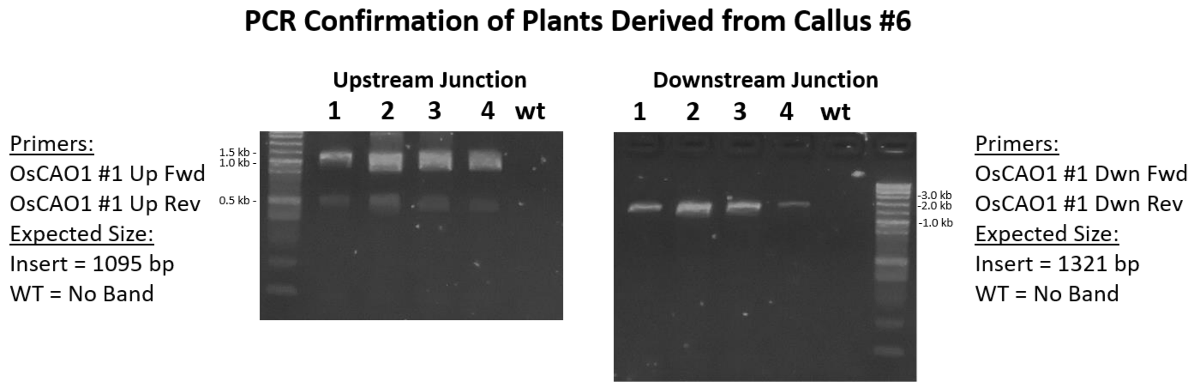

Figure S9: PCR amplification of the upstream (primer pair P1:OsCAO1#1 Up Fwd and P2:OsCAO1#1 Up Rev) and downstream (primer pair P3:OsCAO1#1 Dwn Fwd and P4:OsCAO#1 Dwn Rev) junction regions to confirm that the plants regenerated from GE0001 callus #6 contain the targeted insertion. Four plants derived from callus #6 along with a wild type (WT) control were screened via the two PCR assays.

S10: Absorption Spectrum of Homozygous Line from GE0001

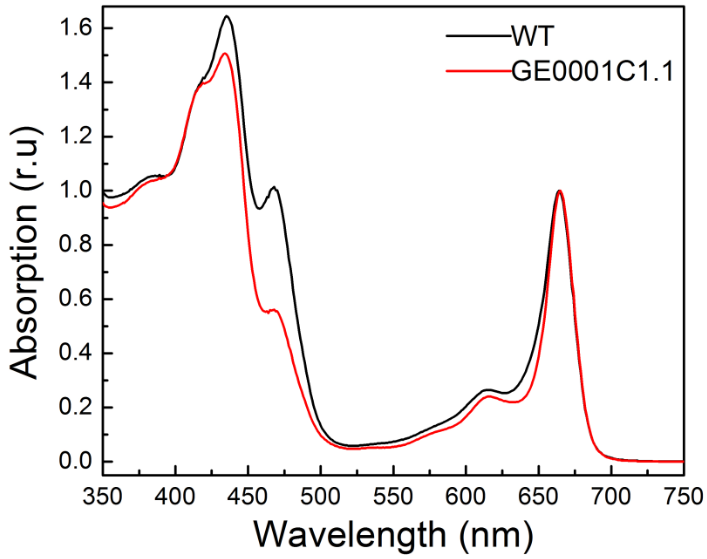

Figure S10: Pigments analysis of homozygous CAO1 genome edited and wild type (WT) plants. The spectra of WT (black) and genome-edited (GE0001C1.1, red) in methanol are normalized at their Qy absorption maxima (660nm) respectively. Note significant decreases in GE0001C1.1 absorbance at 465nm and 650nm, characteristic of Chl*b* absorption spectrum, in the edited plant relative to WT.

S11: Sequence Alignment of GE0046 Insertions

Upstream Arm PCR Sequence Alignment

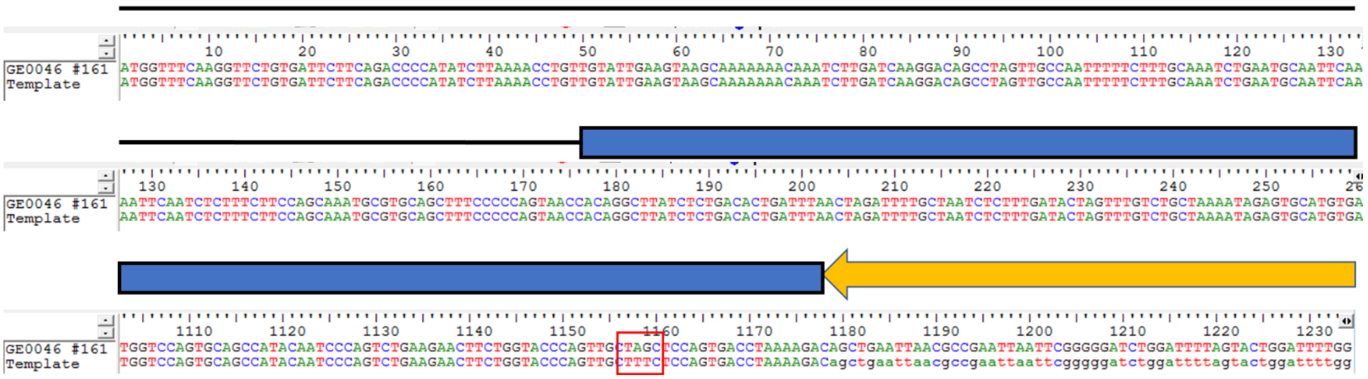

Figure S11: Alignment of upstream junction PCR amplicon of GE0046 callus #161. Genomic sequence is shown as a black line, homology arm shown as blue rectangle, and the hpt marker is shown as a yellow arrow. The targeted mutation of the PAM site (TTTC to TAGC) is outlined in red box.

S12: T7EI assay for GE0001

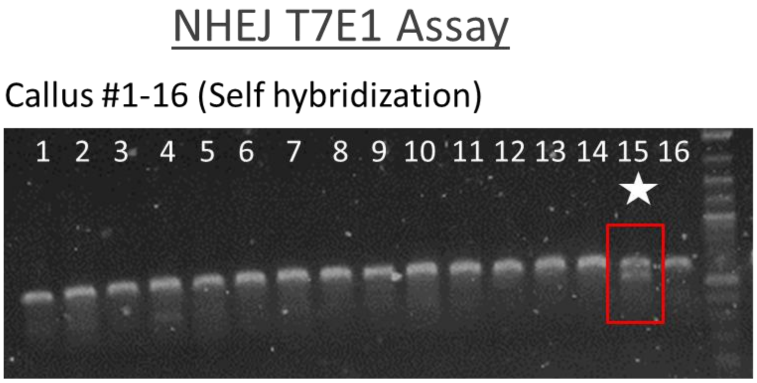

Figure S12: Calli were screened via T7EI assay for indels from GE0001 calli. The experiment was run with self-hybridization of PCR products. Callus #15 showed a positive result in the T7EI assay. Primers' (OsCAO1 #1 Fwd and OsCAO1 #2 Rev) sequences are listed in Table S1.

S13: T7EI Assay for GE0031

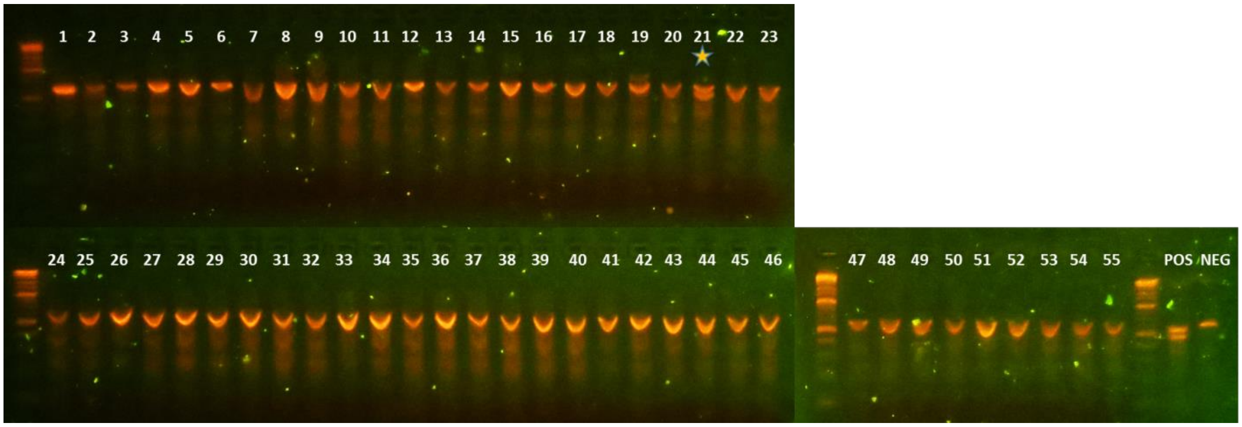


```
WT CAO1 Site #1   : CACCTTTCTGGAGCAACACCTGAAGGAAGGCTTGATGAG//ATGGATGC
GE0031 Callus #21: CACCTTTCTGGAGCAACACCTGAA----AACAAA---//--ATGGATGC
```

Figure S13: A T7EI assay was performed on calli # 1-55 from GE0031 with positive (POS) and negative (NEG) controls shown at the bottom right. One callus piece (#21, starred) was shown to be positive for an indel. The sequence of the indel was verified in the bottom panel (PAM site in blue; sequence targeted by gRNA in red and underlined; // denotes 37 bp sequence not shown to allow for alignment of a large deletion). The indel contained a large 52 bp deletion with a small 6 bp insertion of unknown origin.

S14: Sequence Analysis of Plants Regenerated from Experiment GE0046

Sequences from GE0046-40

```
Sample      PAM      Target Sequence            Indel
WT      TTGCTTTCTCCAGTGACCTAAAAGACGATACAATGGTA
40-1    TTGCTTTCTCCAGTGACCTAAAAGACGATACAATGGTA   x
40-2    TTGCTTTCTCCAGTGACCTAA---------CAATGGTA   -9
40-3    TTGCTTTCTCCAGTGACCTA------------ATGGTA   -11
40-4    TTGCTTTCTCCAGTGACCTA----------CAATGGTA   -10
```

Sequences from GE0046-62

```
Sample      PAM      Target Sequence            Indel
WT      TTGCTTTCTCCAGTGACCTAAAAGACGATACAATGGTA
62-1    TTGCTTTCTCCAGTGACCTAAA--------CAATGGTA   -8
62-2    TTGCTTTCTCCAGTGACCTAA---------CAATGGTA   -9
62-3    TTGCTTTCTCCAGTGACCTAAA--------CAATGGTA   -8
```

Sequences from GE0046-90

```
Sample      PAM      Target Sequence            Indel
WT      TTGCTTTCTCCAGTGACCTAAAAGACGATACAATGGTA
90-1    TTGCTTTCTCCAGTGACCTAAA--------CAATGGTA   -8
90-2    TTGCTTTCTCCAGTGACCTAAA--------CAATGGTA   -8
```

Figure S14: Sequence alignments of CAO1 site #2 amplicons from regenerated plants derived from experiment GE0046 indel positive calli. Amplicons were sub-cloned and sequenced via Sanger sequencing.
